# Sexual function in women with complex PTSD: a comparative study

**DOI:** 10.3389/fpsyt.2026.1796972

**Published:** 2026-06-12

**Authors:** Rodrigo Ramirez-Rodriguez, Ángel Alberto Puig-Lagunes, Rafael Fernández-Demeneghi, Ana Karina Ceja-Venegas, Yuliana Yessy Gomez-Rutti, Miriam Tecamachaltzi-Silvarán, Isidro Vargas-Moreno, Alma Gabriela Martínez-Moreno, León Germán-Ponciano, Gilberto Uriel Rosas-Sanchez

**Affiliations:** 1 Instituto Politécnico Nacional, Ciudad de Mexico, Mexico; 2 Facultad de Medicina, Universidad Veracruzana, Minatitlán, Veracruz, Mexico; 3 Instituto de Investigaciones en Comportamiento Alimentario y Nutrición, Universidad de Guadalajara, Ciudad Guzmán, Mexico; 4 Instituto Colimense de Ciencias Forenses, Colima, Mexico; 5 Universidad Privada del Norte, Lima, Peru; 6 Facultad de Ciencias en Desarrollo Humano, Universidad Autónoma de Tlaxcala, Tlaxcala, Mexico; 7 Laboratorio de Neurofarmacología, Instituto de Neuroetología, Universidad Veracruzana, Xalapa, Veracruz, Mexico; 8 Departamento de Ciencias de la Tierra y la Vida, Centro Universitario de Los Lagos, Lagos de Moreno, Jalisco, Mexico

**Keywords:** complex ptsd, Mexico, PTSD, sexual function, women

## Abstract

**Background:**

Posttraumatic stress disorder (PTSD) is associated with impaired sexual functioning in women, but the effects of complex PTSD (CPTSD) remain unclear. This study tested three hypotheses: (1) women with CPTSD would report lower overall sexual function than women with PTSD and trauma-exposed controls; (2) the pain domain would show the largest group differences; and (3) CPTSD symptom severity would be negatively associated with sexual function, while PTSD symptom severity would not.

**Methods:**

A cross-sectional study included 386 Mexican female university students (18–55 years) who completed the Female Sexual Function Index (FSFI), International Trauma Questionnaire (ITQ), and Adverse Childhood Experiences Questionnaire (ACE-IQ). Group differences were assessed using ANOVA with *post-hoc* comparisons. Multivariable linear regressions examined whether CPTSD severity predicted sexual function independently of PTSD severity and age. A sensitivity analysis excluded women aged 45 and older.

**Results:**

FSFI total scores differed significantly across groups (F = 3.52, p = 0.031). Women with CPTSD reported lower overall sexual function (M = 26.56, SD = 6.41) than trauma-exposed controls (M = 28.86, SD = 5.03; p = 0.047). In the pain domain, women with CPTSD reported greater sexual pain than controls (F = 6.35, p = 0.002). Multivariable regressions showed that CPTSD severity predicted lower FSFI total scores (β = -0.22, p < 0.001), independent of age and PTSD severity (adjusted R^2^ = 0.028). For sexual pain, the bivariate association with CPTSD (rho = -0.16, p < 0.01) did not persist after adjusting for age; age was the only significant predictor (β = 0.02, p = 0.007). Sensitivity analyses yielded unchanged results.

**Conclusions:**

CPTSD severity is associated with poorer overall sexual function, independent of age and PTSD severity. However, the association with sexual pain did not persist after accounting for age. Clinical and research implications are discussed.

## Introduction

Traumatic experiences substantially increase the risk of developing posttraumatic stress disorder (PTSD), a psychiatric condition with an estimated global lifetime prevalence of approximately 5.6% (1). PTSD is characterized by intrusive re-experiencing of traumatic events, avoidance of trauma-related stimuli, and persistent hyperarousal, symptoms that can significantly disrupt emotional, cognitive, and interpersonal functioning. Among individual risk factors, gender plays a critical role, as women consistently demonstrate a higher vulnerability to PTSD compared to men (2). This gender difference is well-documented in community and non-veteran populations, with meta-analytic estimates indicating that women are approximately twice as likely as men to meet PTSD criteria at some point in their lives (3).

Beyond its psychological and emotional consequences, PTSD has been shown to interfere with multiple domains of interpersonal functioning, including sexual health. In women, core PTSD symptoms—such as hyperarousal, emotional numbing, avoidance, and intrusive memories—may directly compromise sexual responsiveness and intimacy. Previous research has consistently linked PTSD in women to reductions in sexual desire, arousal, lubrication, orgasm, and satisfaction, as well as increased experiences of genital pain (4). A systematic review of 43 studies confirmed that PTSD is associated with increased risk of sexual difficulties, particularly in the domains of sexual desire, satisfaction, and distress (5). In more severe cases, PTSD has been associated with diagnosable sexual dysfunctions, including hypoactive sexual desire disorder, sexual arousal disorder, and orgasmic disorder (6, 7). These findings underscore the relevance of sexual functioning as an important, yet often overlooked, domain of trauma-related impairment.

Advances in trauma-related diagnostic frameworks have further refined the conceptualization of posttraumatic psychopathology. In 2018, the World Health Organization introduced the eleventh revision of the International Classification of Diseases (ICD-11), formally recognizing complex posttraumatic stress disorder (CPTSD) as a diagnosis distinct from PTSD (8). While CPTSD includes the core PTSD symptom clusters—re-experiencing, avoidance, and a persistent sense of threat—it is additionally characterized by pervasive disturbances in self-organization. These include affect dysregulation, a persistent negative self-concept, and chronic disturbances in interpersonal relationships.

Unlike PTSD, which may arise following a single or circumscribed traumatic event, CPTSD has been strongly linked to cumulative and prolonged adverse childhood experiences, particularly those occurring within caregiving and interpersonal contexts (9). Exposure to chronic trauma during early developmental periods is thought to exert enduring effects on emotional regulation, relational capacity, and self-perception across the lifespan. Similar to PTSD, gender represents a significant risk factor for CPTSD, with women being disproportionately affected (10). Consequently, sustained trauma exposure during childhood and adolescence may heighten women’s vulnerability to long-term psychopathological outcomes, including impairments in intimate and sexual functioning.

Despite a growing body of research examining sexual functioning among women with PTSD, the relationship between CPTSD and female sexual function remains largely unexplored. To date, no empirical studies have specifically examined how CPTSD symptomatology relates to sexual functioning across its multiple domains. This gap in the literature is particularly notable given the central role that affect regulation, self-concept, and interpersonal functioning—core features of CPTSD—play in sexual health and intimacy.

In Mexico, empirical research on CPTSD is still in its early stages. A recent study examined body esteem in Mexican women with CPTSD, finding that CPTSD was associated with significantly diminished body esteem compared to both PTSD and trauma-exposed controls (11). However, to our knowledge, no published studies have specifically investigated sexual functioning in women with CPTSD in Mexico, and research from other Latin American countries remains extremely limited. This scarcity of research limits our understanding of how complex trauma affects sexual health in culturally distinct contexts where traditional gender norms (12), socioeconomic inequalities, and limited access to specialized sexual health care (13, 14) may shape both trauma expression and sexual well-being.

This gap is further pronounced in Latin American contexts, including Mexico, where complex sociocultural conditions shape women’s complex sexual health. Traditional gender norms, persistent socioeconomic inequalities, and patriarchal attitudes continue to influence women’s experiences of sexuality and bodily autonomy (15). These factors are often compounded by economic disadvantage, which limits many women’s access to health-related resources and education (16). It is important to note that these sociocultural variables are provided as contextual background for interpreting the findings, rather than as directly measured predictors in the current analyses. Future studies should directly assess these factors to examine their role in trauma-related sexual dysfunction. In contrast to high-income settings where psychological and sexual health services may be more accessible, Mexican women may experience trauma and its sexual health consequences within environments characterized by structural constraints and limited specialized care. Such contextual conditions may influence not only the expression of trauma-related symptoms but also their recognition and clinical management.

Anchoring the study of CPTSD and sexual functioning within this national context is therefore essential for developing a more nuanced understanding of women’s trauma-related experiences. Examining these associations in a Mexican sample contributes culturally informed evidence to the international literature and addresses an important gap in trauma and sexual health research.

Against this backdrop, the present study tested three specific hypotheses (1): women with CPTSD would report significantly lower overall sexual function compared to women with PTSD and trauma-exposed controls (2); the sexual pain domain would show the largest magnitude of difference between CPTSD and the other two groups; and (3) CPTSD symptom severity would be negatively associated with sexual function, while PTSD symptom severity would not show a significant association after accounting for CPTSD symptoms. By focusing on sexual function as a central outcome, this study seeks to enhance the understanding of CPTSD and to underscore the importance of incorporating sexual health into trauma-informed assessment and care.

## Methods

### Study design

A cross-sectional, observational, and comparative study was conducted between March and April 2025, targeting undergraduate and graduate students in Mexico. The study aimed to examine sexual functioning in women with PTSD or CPTSD trauma-related symptoms.

### Sample

Participants aged 18 to 55 years were recruited through de advertisements in university communities and research institutions using a non-probabilistic sampling method. Inclusion criteria required participants to be cisgender women of Mexican nationality, aged 18 years or older, and sexually active within the past four weeks. Exclusion criteria included being sexually inactive during the past four weeks, identifying as non-cisgender, non-Mexican nationality, being under the age of 18, or having a diagnosed mental disability. These criteria yielded a sample of 386 participants.

### Data collection

The study was conducted in accordance with the Declaration of Helsinki, and approved by the Institutional Review Board Research Ethics Committee of Faculty of Medicine, Minatitlan Campus at Universidad Veracruzana (F-001-CI-2025, Approved date: 25 February 2025) for studies involving humans. Participants were contacted by members of the research team to explain the rationale and purpose of the study. Information about the study was disseminated via WhatsApp, Facebook, and Instagram. Participants’ anonymity and confidentiality were assured. Electronic informed consent was obtained, and responses to the research instruments were collected using an online survey administered through Google Forms.

## Measures

### Sociodemographic data

The survey included questions about sociodemographic data, such as age, area of residence, education level, income level, employment status, and maternal status (i.e., whether the respondent has children).

### International trauma questionnaire

The International Trauma Questionnaire (ITQ) was administered to assess the presence of PTSD and CPTSD. The ITQ has demonstrated adequate psychometric properties for use with the Mexican population (17). A PTSD diagnosis requires experiencing at least one symptom from two of the following three categories: (1) re-experiencing the trauma in the present moment, (2) avoidance behaviors, and (3) a heightened sense of current threat. Additionally, at least one associated functional impairment must be reported. A symptom or impairment is considered present if it receives a score greater than two. CPTSD includes all the criteria for PTSD, but also requires the presence of at least one symptom from each of the three PTSD symptom clusters mentioned above. Furthermore, a CPTSD diagnosis involves additional symptoms grouped under disturbances in self-organization (DSO), including: (1) emotional dysregulation, (2) a negative self-perception, and (3) disturbances in relationships. To meet the criteria for CPTSD, there must also be evidence of functional impairment related to both the PTSD and DSO symptoms. Each impairment must also score above two. Importantly, a person can be diagnosed with either PTSD or CPTSD, but not both. If the CPTSD criteria are met, only the CPTSD diagnosis is applied. Following the guidelines of the ITQ, participants were classified into three groups: trauma-exposed controls (n = 306; 79.27%), PTSD (n = 42; 10.88%), and CPTSD (n = 38; 9.84%). In this study, the internal consistency of the ITQ was good (α = 0.81).

### Female sexual function index

The Female Sexual Function Index (FSFI) was used to evaluate sexual functioning, which has demonstrated robust psychometric properties in the Mexican population (18). The FSFI is a 19-item self-report questionnaire that evaluates six domains of sexual function: desire (2 items), arousal (4 items), lubrication (4 items), orgasm (3 items), satisfaction (3 items), and pain (3 items, reverse-scored). Each item is rated on a 5-point Likert scale. Following established scoring guidelines, a total FSFI score of 26.55 or lower is indicative of sexual dysfunction. In this study, the internal consistency of FSFI was good (α = 0.83).

### Adverse childhood experiences – international questionnaire

The Adverse Childhood Experiences – International Questionnaire (ACE-IQ) was administered to assess adverse childhood experiences that occurred before the age of 18. It has demonstrated adequate psychometric properties in a sample of Mexican adults (19). For this study, the dichotomous (Yes/No) response format was employed to reduce the complexity of ordinal data and to enhance statistical weighting through binary categorization. This self-administered instrument comprises 31 items across five modules: family violence (6 items), sexual abuse (4 items), peer victimization (7 items), family dysfunction (7 items), and community violence (7 items). Higher total scores reflect a greater frequency of diverse adverse experiences. In this study, the internal consistency of ACE-IQ was acceptable (α = 0.73).

### Statistical analyses

Data analysis and graphs were performed using RStudio (20) for Macintosh. Categorical variables were described using absolute and relative frequencies, whereas quantitative variables were summarized as means and standard deviations. Differences in sexual function among diagnostic groups were assessed using one-way analysis of variance. *Post hoc* comparisons were conducted using Tukey’s test. Associations between sexual functioning and trauma-related symptoms were examined using Spearman correlations of FSFI and ITQ domain and total scores. Multivariable linear regression analyses were conducted to examine whether CPTSD severity explained unique variance in sexual functioning above and beyond PTSD severity and age. The first model used the FSFI total score as the dependent variable, with PTSD severity, CPTSD severity, and age entered as simultaneous predictors. Subsequent separate models were run for each FSFI domain (desire, arousal, lubrication, orgasm, satisfaction, and pain) using the same set of predictors. To address concerns about potential confounding by perimenopausal status, a sensitivity analysis was performed excluding women aged 45 and older (n = 11, 2.8% of the sample). All primary analyses (ANOVA and multivariable regressions) were repeated on this subsample. All statistical analyses were two-tailed, and statistical significance was defined as p < 0.05.

## Results

### Sociodemographic characteristics and adverse childhood experiences of the sample

The mean age of participants was 26.39 years (SD = 7.82). Most participants were in the 18–25 age group (61.7%), had a bachelor’s degree (82.9%), lived in urban areas (88.9%), reported having a paid job (55.4%), and did not have children (76.7%). The most frequently reported income category was below MX $12,977 (32.6%). Regarding adverse childhood experiences, participants reported family dysfunction (90.9%), peer violence (82.1%), family violence (76.9%), community violence (59.3%), and sexual victimization (44.8%). Among participants with cumulative ACE exposure, the highest proportion reported exposure to five ACE domains (30.6%). These percentages represent the observed descriptive statistics in our community sample and are not interpreted as population estimates. A detailed overview of the sample characteristics is presented in **Table 1**.

**Table 1:** Sociodemographic characteristics and childhood adversity of the sample (n = 386).

Variable	Level	n (%) or mean (sd)
Age (years)	—	26.39 (7.82)
	18-25	238 (61.7%)
	26-35	95 (24.6%)
	36-45	42 (10.9%)
	46-55	11 (2.8%)
Studies	Bachelor	320 (82.9%)
	Postgraduate	66 (17.1%)
Residence	Rural	43 (11.1%)
	Urban	343 (88.9%)
Income	< MX $12, 977	126 (32.6%)
	< MX $18, 569	25 (6.5%)
	< MX $23, 451	124 (32.1%)
	< MX $77, 975	57 (14.8%)
	< MX $9, 313	54 (14.0%)
Job	No	172 (44.6%)
	Yes	214 (55.4%)
Children	No	296 (76.7%)
	Yes	90 (23.3%)
Family violence	Yes	297 (76.9%)
	No	89 (23.1%)
Sexual victimization	Yes	173 (44.8%)
	No	213 (55.2%)
Peer violence	Yes	317 (82.1%)
	No	69 (17.9%)
Family dysfunction	Yes	351 (90.9%)
	No	35 (9.1%)
Community violence	Yes	229 (59.3%)
	No	157 (40.7%)
Cumulative ACE domains		
	0	11 (2.8%)
	1	31 (8.0%)
	2	42 (10.9%)
	3	74 (19.2%)
	4	110 (28.5%)
	5	118 (30.6%)

### Sexual function across trauma control, PTSD, and CPTSD groups

**Figure 1:**
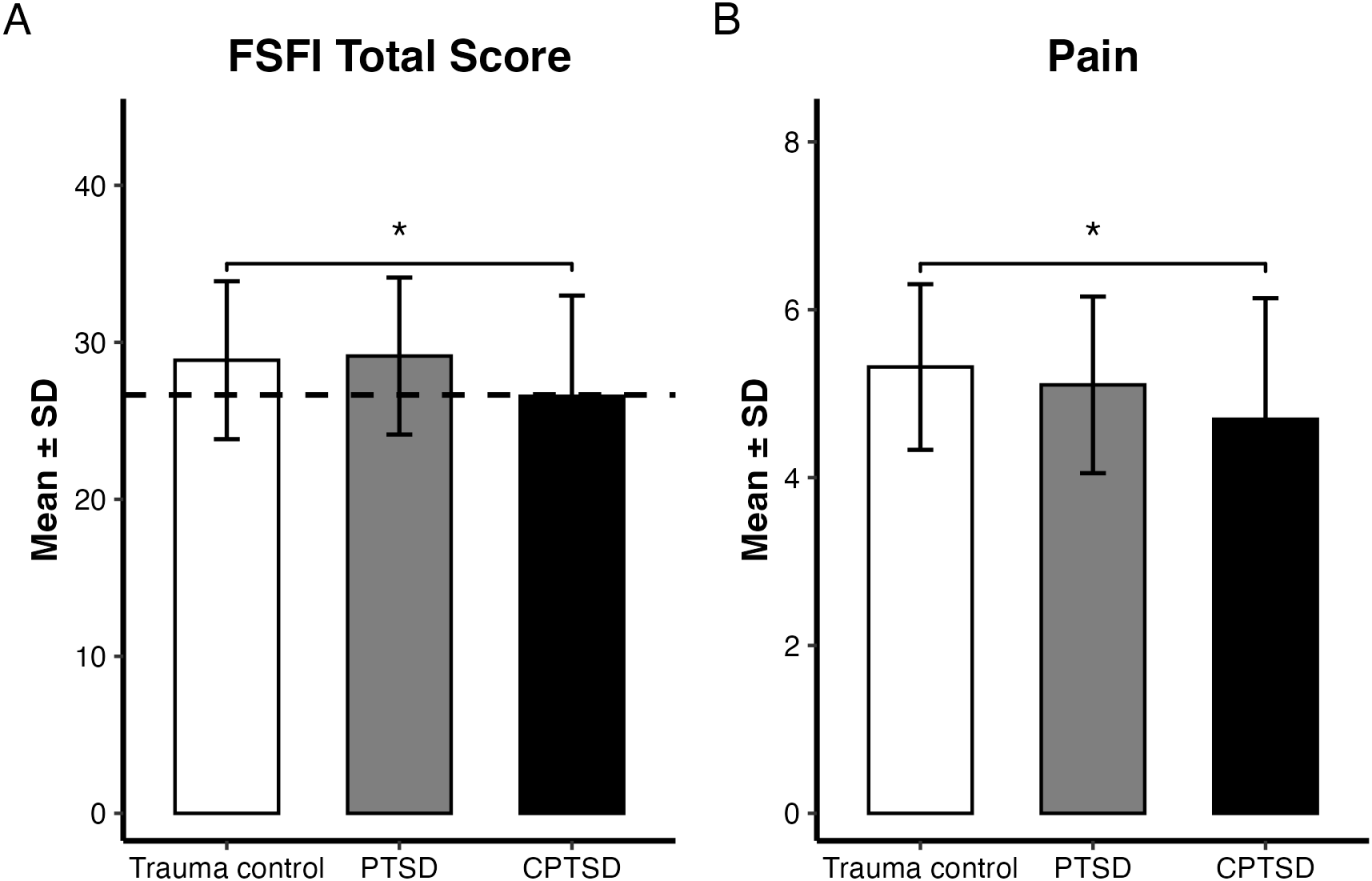
**(A)** Mean FSFI Total Score (± SD) across groups. The dashed line indicates the cutoff score for sexual dysfunction (26.55). **(B)** Mean pain scores (± SD) across groups. Asterisk denotes statistically significant group differences (p < 0.05).

Mean scores for sexual function domains were compared across the trauma control, PTSD, and CPTSD groups, as detailed in **Table 2**. One-way ANOVA revealed significant group differences in the total FSFI score (F = 3.52, p = .031). *Post hoc* analyses indicated that women in the CPTSD group reported significantly lower total sexual function (M = 26.56, SD = 6.41) compared to the trauma control group (M = 28.86, SD = 5.03), while no significant differences were observed between the PTSD and trauma control groups, as shown in **Figure 1A**.

**Table 2:** Means and standard deviations of FSFI scores by diagnosis.

Variable	Trauma control (n = 306)	PTSD (n = 42)	CPTSD (n = 38)	F	*p*
Arousal	4.75 (1.16)	5.01 (1.00)	4.58 (1.34)	1.40	0.247
Desire	4.03 (1.23)	4.21 (1.26)	3.79 (1.42)	1.16	0.316
Lubrication	5.18 (0.98)	5.17 (1.05)	4.82 (1.27)	2.21	0.111
Orgasm	4.56 (1.35)	4.57 (1.45)	4.16 (1.41)	1.48	0.228
Pain	5.32 (0.99) a	5.10 (1.05) ab	4.69 (1.44) b	6.35	0.002*
Satisfaction	5.02 (1.19)	5.06 (1.14)	4.53 (1.33)	2.91	0.056
Total score	28.86 (5.03) a	29.13 (5.00) ab	26.56 (6.41) b	3.52	0.031*

Different letters indicate statistically significant differences in multiple pairwise comparisons.

Significant group differences were also found for the pain domain (F = 6.35, p = .002). *Post hoc* comparisons showed that the CPTSD group reported significantly lower pain scores, indicating greater sexual pain (M = 4.69, SD = 1.44) than the trauma control group (M = 5.32, SD = 0.99). No other pairwise comparisons reached statistical significance, as shown in **Figure 1B**.

Regarding sexual dysfunction classification, 26.5% of participants in the trauma control group (81/306), 39.5% in the CPTSD group (15/38), and 19.0% in the PTSD group (8/42) were classified as having sexual dysfunction.

### Sensitivity analysis excluding women aged 45 and older

To address concerns about potential confounding by perimenopausal status, we conducted a sensitivity analysis excluding women aged 45 and older (n = 11, 2.8% of the original sample). All primary analyses were repeated on this subsample (n = 375). The pattern of results remained unchanged: the one-way ANOVA comparing FSFI total scores across diagnostic groups remained significant, F(2, 369) = 3.77, p = 0.02, and CPTSD severity remained a significant predictor of lower FSFI total scores in the multivariable regression model (β = -0.21, SE = 0.06, p = 0.002). These analyses confirm that the inclusion of perimenopausal women did not drive the observed findings.

### Associations between sexual functioning and trauma-related symptoms

**Figure 2:**
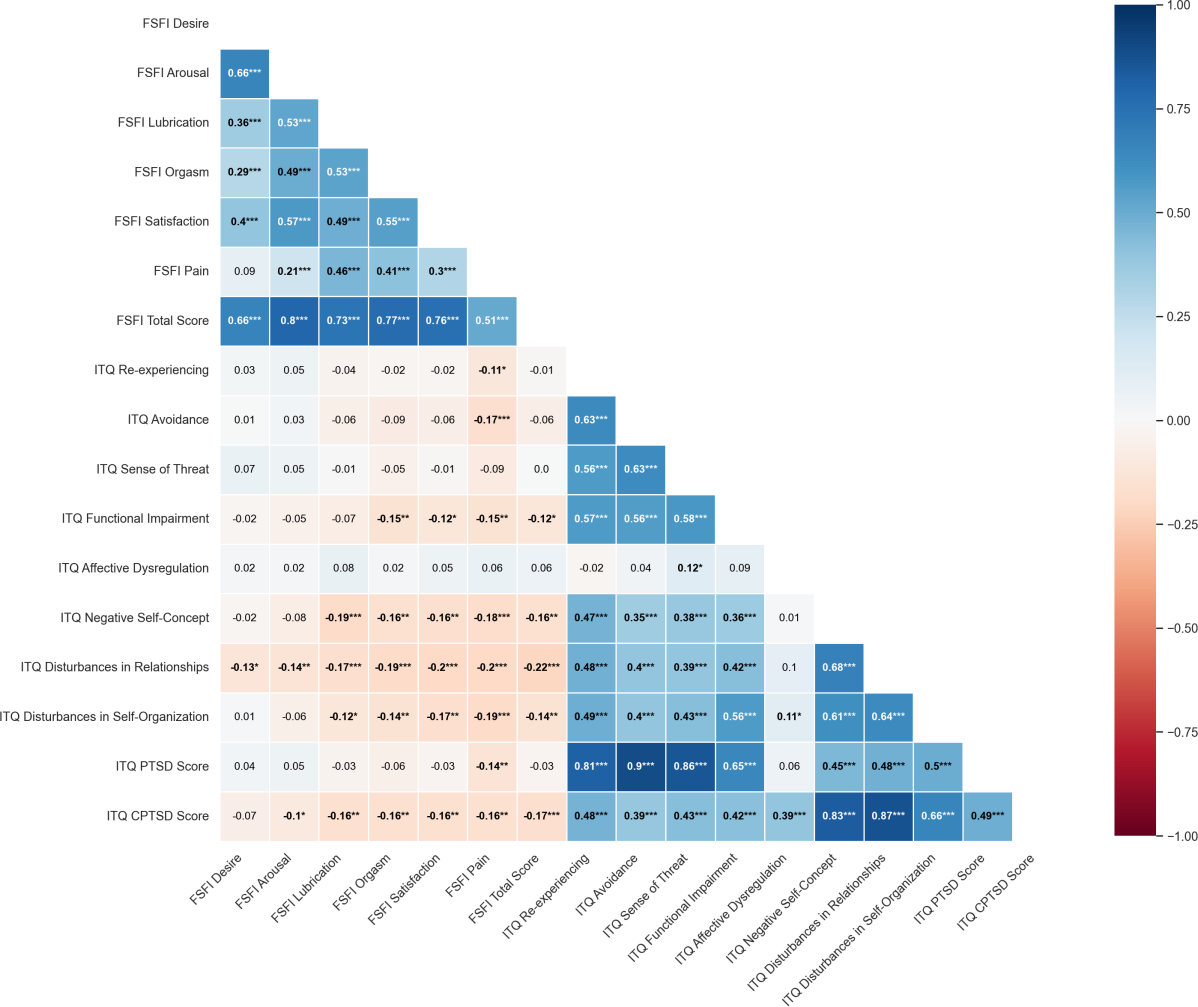
Spearman correlations between sexual functioning and trauma-related symptoms. *p < 0.05, **p <0.01, ***p < 0.001.

Significant correlations were observed between trauma symptom severity and sexual functioning, as shown in **Figure 2**. The FSFI total score was not significantly associated with PTSD symptom severity (rho = −.03, p >.05). However, CPTSD symptom severity showed a small but statistically significant negative association with overall sexual functioning, such that higher CPTSD total scores were associated with lower FSFI total scores (rho = −.17, p <.001).

Sexual pain was differentially associated with trauma symptom severity. FSFI Pain scores were significantly and negatively associated with both PTSD and CPTSD symptom severity, with stronger associations observed for CPTSD. Specifically, higher PTSD total scores were associated with greater sexual pain (rho = −.14, p <.01), whereas higher CPTSD total scores showed a stronger negative association with FSFI Pain scores (rho = −.16, p <.01). All correlation coefficients fell within the weak-to-modest range, indicating small effect sizes.

### Multivariable linear regression analyses

To formally test whether CPTSD symptoms explain unique variance in sexual functioning above and beyond PTSD symptoms and age, we conducted a multivariable linear regression analysis with FSFI total score as the dependent variable and PTSD severity, CPTSD severity, and age as simultaneous predictors. The overall model was significant F(3, 382) = 4.70, p = 0.003, adjusted R^2^ = 0.028. CPTSD severity emerged as a significant negative predictor of FSFI total score (β = -0.22, SE = 0.07, p <.001), indicating that higher CPTSD symptom severity was associated with lower overall sexual functioning. In contrast, PTSD severity (β = 0.03, SE = 0.05, p = 0.52) and age (β = 0.02, SE = 0.03, p = 0.52) were not significant predictors.

We then conducted separate multivariable regression models for each FSFI domain: desire, arousal, lubrication, orgasm, satisfaction, and pain. The results are presented in **Table 3**. CPTSD severity was a significant negative predictor across five domains: desire (β = -0.03, SE = 0.02, p = 0.04), arousal (β = -0.04, SE = 0.01, p = 0.006), lubrication (β = -0.04, SE = 0.01, p = 0.002), orgasm (β = -0.05, SE = 0.02, p = 0.005), and satisfaction (β = -0.04, SE = 0.02, p = 0.004). For FSFI pain, neither CPTSD severity (β = -0.02, SE = 0.01, p = 0.182) nor PTSD severity (β = -0.02, SE = 0.01, p = 0.059) reached statistical significance. Age was the only significant predictor in the pain model (β = 0.02, SE = 0.01, p = 0.007), with older women reporting less sexual pain. The bivariate association between CPTSD severity and pain (rho = -0.16, p < 0.01) did not remain significant after adjusting for age and PTSD severity.

**Table 3:** Multivariable linear regression models predicting FSFI Total and domain scores.

	Predictor	β	SE	p	95% CI	Model R^2^ (adj)
FSFI Total Score	PTSD severity	0.03	0.05	0.526	[-0.07, 0.14]	0.028
	CPTSD severity	-0.22	0.07	**<.001**	[-0.35, -0.09]	
	Age	0.02	0.03	0.520	[-0.04, 0.09]	
FSFI Pain	PTSD severity	-0.02	0.01	0.059	[-0.04, 0.00]	0.043
	CPTSD severity	-0.02	0.01	0.182	[-0.04, 0.01]	
	Age	0.02	0.01	**0.007**	[0.01, 0.03]	
FSFI Desire	PTSD severity	0.02	0.01	0.157	[-0.01, 0.04]	0.004
	CPTSD severity	-0.03	0.02	**0.042**	[-0.06, -0.00]	
	Age	0.00	0.01	0.746	[-0.02, 0.01]	
FSFI Arousal	PTSD severity	0.02	0.01	0.077	[-0.00, 0.04]	0.013
	CPTSD severity	-0.04	0.01	**0.006**	[-0.07, -0.01]	
	Age	0.00	0.01	0.788	[-0.02, 0.01]	
FSFI Lubrication	PTSD severity	0.00	0.01	0.759	[-0.02, 0.02]	0.023
	CPTSD severity	-0.04	0.01	**0.002**	[-0.07, -0.01]	
	Age	0.00	0.01	0.761	[-0.01, 0.02]	
FSFI Orgasm	PTSD severity	0.00	0.01	0.846	[-0.02, 0.03]	0.046
	CPTSD severity	-0.05	0.02	**0.005**	[-0.08, -0.01]	
	Age	0.03	0.01	**0.003**	[0.01, 0.04]	
FSFI Satisfaction	PTSD severity	0.01	0.01	0.487	[-0.02, 0.03]	0.028
	CPTSD severity	-0.04	0.02	**0.004**	[-0.07, -0.01]	
	Age	-0.02	0.01	**0.010**	[-0.04, -0.00]	

Bold values denotes statistically significant differences (p < 0.05).

## Discussion

The present study examined sexual functioning in women with CPTSD, providing initial evidence of associations with impairments in overall sexual function and, in particular, heightened pain among a Mexican community sample. To our knowledge, this is the first study to specifically document associations between CPTSD and alterations in sexual functioning within the pain domain among women with CPTSD in Mexico. These findings extend previous research on PTSD, which consistently demonstrates that women with PTSD experience reductions across multiple domains of sexual functioning alongside increased genital pain (4). In line with prior evidence, women with PTSD who were exposed to childhood sexual abuse have been shown to report significantly poorer sexual functioning compared to women without PTSD (21). A history of sexual abuse, particularly during childhood and adolescence, is associated not only with PTSD but also with gynecological sequelae, including dyspareunia and chronic pelvic pain, independent of PTSD diagnosis (22). Our correlation analyses indicated a small but significant negative association between FSFI total score and CPTSD symptom severity, whereas no significant correlation was found with PTSD symptom severity. The observed effect sizes (rho ranging from -0.10 to -0.22) were weak to modest, indicating that, while statistically significant, the clinical significance of these associations should be interpreted cautiously. These results suggest that higher CPTSD symptomatology may be associated with greater sexual dysfunction, supporting the notion that complex trauma may exert broader effects on women’s sexual health than PTSD alone. However, the cross-sectional design precludes any causal inferences.

The multivariable regression analyses revealed that CPTSD severity remained a significant predictor of lower FSFI total scores and four of the five domain scores (desire, arousal, lubrication, satisfaction) after controlling for PTSD severity and age. Notably, the association between CPTSD and sexual pain observed in bivariate analyses did not persist in the multivariable model, where age emerged as the only significant predictor. This suggests that the relationship between CPTSD and sexual pain did not persist after accounting for age, with younger women reporting both higher CPTSD symptoms and more sexual pain.

Taken together, these findings highlight the importance of the developmental timing and cumulative nature of trauma exposure in shaping long-term sexual health outcomes. In the context of CPTSD, which is closely associated with chronic interpersonal trauma during early life, such exposures may contribute to persistent sexual difficulties, with pain emerging as a particularly salient aspect of sexual dysfunction. Domain-specific analyses further indicated that pain was the sexual functioning domain most consistently associated with CPTSD symptom severity. Prior research has consistently identified pain as a central contributor to female sexual dysfunction (23). Importantly, among various forms of childhood victimization, sexual abuse shows the strongest association with genital pain in sexually active women (24). Therefore, the elevated sexual pain reported by women with CPTSD in our sample may be closely related to early adverse experiences, particularly sexual victimization.

Neurobiological evidence offers potential insight into the mechanisms underlying these sexual difficulties. Neuroimaging studies indicate that individuals with CPTSD exhibit hyperactivation in brain regions involved in emotional and interoceptive processing, notably the amygdala and insular cortex, in response to emotionally salient stimuli (25). The insular cortex is critically involved in nociceptive processing (26), while the amygdala contributes to the emotional appraisal of nociceptive stimuli (27). Altered functioning in these circuits may amplify pain perception and negatively affect sexual experiences by modifying both emotional evaluation and interoceptive awareness. In addition, decreased thalamic activity has been observed in individuals with CPTSD during cognitive inhibition tasks (28). Given the thalamus’s role as a central hub for sensory integration (29) and its vulnerability to adverse childhood experiences (30), disruptions in thalamic processing may further impair accurate perception of bodily and environmental cues, including sexual stimuli. Together, these neurobiological alterations provide a plausible neurobiological framework linking early trauma exposure, CPTSD, and sexual pain, though direct evidence linking these mechanisms to sexual function specifically is still needed.

Interestingly, no significant differences in sexual functioning or its domains were observed between the PTSD group and either the CPTSD or trauma control groups. This aligns with prior research showing that women with PTSD do not always present sexual dysfunction, and suggests that the nature of the trauma may be more influential than the diagnostic label itself (31). This apparent discrepancy may reflect differences between dimensional associations and categorical group comparisons. Specifically, interpersonal traumas—such as sexual assault, serious accidents, or the death of a loved one—have been identified as particularly strong predictors of both PTSD onset and symptom severity (32, 33). Moreover, the type and accumulation of childhood trauma increase the likelihood of a CPTSD diagnosis (34). These findings suggest that sexual dysfunction in women with trauma-related psychiatric disorders may not be solely attributable to the diagnostic category, but rather to the specific nature, timing, and chronicity of traumatic experiences, which appear particularly impactful in CPTSD.

### Implications

The present findings, while cross-sectional and based on weak-to-moderate effect sizes, suggest several clinically relevant considerations. First, sexual pain should be routinely assessed in women presenting with CPTSD symptoms, as this domain showed the strongest bivariate association with CPTSD severity. Second, trauma-informed sex therapy for women with CPTSD may need to address affect regulation and relational disturbances specifically, given that these disturbances in self-organization (DSO) symptoms distinguish CPTSD from PTSD and may uniquely contribute to sexual difficulties. Third, evidence from PTSD treatment research indicates that trauma-focused psychotherapies, such as Cognitive Processing Therapy (CPT), can lead to improvements in sexual distress, with changes in sexual functioning covarying with reductions in PTSD symptoms (35). However, whether these effects extend to CPTSD populations remains unknown. Fourth, dissociation—which is more common in complex trauma presentations—may be a negative prognostic indicator for improvement in sexual distress during treatment (36), suggesting that interventions addressing dissociative symptoms may be particularly relevant for women with CPTSD and sexual dysfunction. Finally, our results emphasize the relevance of early-life and cumulative traumatic experiences in shaping sexual outcomes. Interventions or educational programs aimed at promoting sexual health and well-being could benefit from incorporating trauma-informed principles, particularly by acknowledging the timing, nature, and severity of prior trauma. Evidence from PTSD research indicates that targeted interventions can mitigate the impact of trauma on sexual functioning (37, 38). While these approaches have demonstrated efficacy in CPTSD populations (39, 40), their impact on sexual functioning has not been specifically examined. Addressing this gap may inform future prevention and support strategies aimed at improving sexual well-being among individuals with CPTSD.

### Strengths, limitations, and future research

This study has several strengths. First, it represents the first comparative analysis of sexual functioning in women with CPTSD, offering novel insights into how trauma-related disorders differentially associate with female sexual health. Second, the study is culturally specific: it is the first to assess sexual functioning in Mexican women with CPTSD using psychometrically validated and culturally adapted instruments. This ensures that the findings are both scientifically robust and contextually relevant, reflecting how trauma, gender norms, and sexuality intersect in Mexican society. By highlighting these dynamics, the study underscores the need for culturally sensitive approaches in understanding and supporting women affected by CPTSD, particularly in contexts where social norms and stigma may exacerbate the impact of trauma on sexual well-being.

Several limitations should be considered. First, the cross-sectional design precludes any causal conclusions regarding the relationship between CPTSD and sexual dysfunction. The associations reported here are correlational only. Second, sexual functioning was assessed via self-report, potentially introducing social desirability bias and underreporting. The sample was predominantly composed of highly educated individuals, restricting generalizability. Third, psychiatric comorbidities were not assessed, despite their known influence on CPTSD, including depression, anxiety, and suicidal ideation. Consequently, it remains unclear whether observed sexual impairments are attributable specifically to CPTSD or to overlapping conditions. Fourth, the observed effect sizes (Spearman rho values between -0.10 and -0.22) were weak to modest, indicating that, while statistically significant, the clinical significance of these associations is uncertain. Fifth, the age range of 18–55 years encompasses premenopausal, perimenopausal, and postmenopausal women, and sexual function varies substantially across these stages. Although our sensitivity analysis excluding women aged 45 and older yielded similar results, future studies should either restrict age ranges or include menopausal status as a covariate. Sixth, the use of a university-based sample, while offering advantages in terms of health literacy and access, limits generalizability to non-student populations. Seventh, the ACE-IQ was used for descriptive purposes only; direct tests of associations between specific ACE domains and sexual function would require a larger sample and a different analytic design.

Future research should explore the role of trauma type—interpersonal versus non-interpersonal—in shaping sexual functioning, as interpersonal traumas may have a stronger impact. Further examination of childhood trauma subtypes, their timing, and cumulative effects is warranted. Studies could also investigate potential mediators or moderators, including resilience, social support, or comorbid conditions, to better understand individual variability in sexual outcomes. Importantly, longitudinal designs are needed to clarify the temporal direction of the association between CPTSD and sexual dysfunction and to identify factors that promote recovery or persistence of difficulties. Finally, adopting an intersectional perspective that considers gender, sexual orientation, and sociocultural context can deepen understanding of how these factors interact with trauma to influence sexual health.

## Conclusions

Our findings indicate that CPTSD symptoms are associated with diminished sexual functioning in women, with sexual pain emerging as a particularly salient feature in bivariate analyses. However, the effect sizes were weak to modest, and the cross-sectional design precludes causal inference. Notably, the association between CPTSD and sexual pain did not remain significant after adjusting for age. These results highlight the importance of considering sexual functioning as an integral dimension in the study of trauma-related symptomatology and underscore the need further to examine this often-overlooked aspect of women’s well-being.

## Data Availability

The datasets for this article are not publicly available due to their sensitive nature. Requests to access the datasets should be directed to the corresponding author.

## References

[1] Kessler RC Aguilar-Gaxiola S Alonso J Benjet C Bromet EJ Cardoso G . Trauma and PTSD in the WHO World Mental Health Surveys. Eur J Psychotraumatol. (2017) 8:1353383. doi: 10.1080/20008198.2017.1353383 29075426 PMC5632781

[2] Tortella-Feliu M Fullana MA Pérez-Vigil A Torres X Chamorro J Littarelli SA . Risk factors for posttraumatic stress disorder: An umbrella review of systematic reviews and meta-analyses. Neurosci Biobehav Rev. (2019) 107:154–65. doi: 10.1016/j.neubiorev.2019.09.013 31520677

[3] McGinty G Fox R Ben-Ezra M Cloitre M Karatzias T Shevlin M . Sex and age differences in ICD-11 PTSD and complex PTSD: An analysis of four general population samples. Eur Psychiatry J Assoc Eur Psychiatr. (2021) 64:e66. doi: 10.1192/j.eurpsy.2021.2239 34602122 PMC8581703

[4] Högbeck I Möller A . Female sexual function six months after sexual assault: post-traumatic stress disorder strongest risk factor for impaired function. J Sex Marital Ther. (2022) 48:112–20. doi: 10.1080/0092623X.2021.1958964 34338163

[5] Bird ER Piccirillo M Garcia N Blais R Campbell S . Relationship between posttraumatic stress disorder and sexual difficulties: A systematic review of veterans and military personnel. J Sex Med. (2021) 18:1398–426. doi: 10.1016/j.jsxm.2021.05.011 34257051 PMC8726013

[6] Meston CM Lorenz TA Stephenson KR . Effects of expressive writing on sexual dysfunction, depression, and PTSD in women with a history of childhood sexual abuse: results from a randomized clinical trial. J Sex Med. (2013) 10:2177–89. doi: 10.1111/jsm.12247 23875721 PMC3775987

[7] O’Loughlin JI Brotto LA . Women’s sexual desire, trauma exposure, and posttraumatic stress disorder. J Trauma Stress. (2020) 33:238–47. doi: 10.1002/jts.22485 32216146

[8] World Health Organization . ICD-11 for Mortality and Morbidity Statistics (2018). Available online at: https://icd.who.int/browse/2025-01/mms/en#585833559 (Accessed January 08, 2026).

[9] Cloitre M . Complex PTSD: assessment and treatment. Eur J Psychotraumatol. (2021) 12:1866423. doi: 10.1080/20008198.2020.1866423 34211640 PMC8221157

[10] Kairyte A Kvedaraite M Kazlauskas E Gelezelyte O . Exploring the links between various traumatic experiences and ICD-11 PTSD and Complex PTSD: A cross-sectional study. Front Psychol. (2022) 13:896981. doi: 10.3389/fpsyg.2022.896981 36186396 PMC9521547

[11] Ramirez-Rodriguez R Puig-Lagunes Á Fernández-Demeneghi R Ceja-Venegas AK Gomez-Rutti YY Tecamachaltzi-Silvarán MB . Body esteem in women with complex PTSD: A comparative study. Psychiatry Int. (2026) 7:46. doi: 10.3390/psychiatryint7020046 30654563

[12] Blanquel-Castro P Juárez-Luna D . The role of social norms in the employment status of Mexican women. Rev Econ. (2025) 42:146–71. doi: 10.33937/reveco.2025.472

[13] Alonso de los Santos MÁ Ramos Tovar ME Alonso de los Santos MÁ Ramos Tovar ME . El derecho a la salud sexual y reproductiva: su accesibilidad desde la interpretación internacional. Cuest Const. (2024) 51. doi: 10.22201/iij.24484881e.2024.51.18161

[14] García-Bello LA Heredia-Pi IB Zavala-Arciniega L Paz-Ballesteros W Velázquez-Viamonte A Serván-Mori E . Care friendliness in adolescent sexual and reproductive health services in Mexico and a characterization of their clients. Int J Health Plann Manage. (2022) 37:204–19. doi: 10.1002/hpm.3512 35661412

[15] Villalobos A Estrada F Hubert C Torres-Ibarra L Rodríguez A Romero I . Sexual and reproductive health among adolescents in vulnerable contexts in Mexico: Needs, knowledge, and rights. PLoS Glob Public Health. (2023) 3:e0002396. doi: 10.1371/journal.pgph.0002396 37910453 PMC10619806

[16] Ramos ME Gibaja-Romero DE Ochoa SA . Gender inequality and gender-based poverty in Mexico. Heliyon. (2020) 6:e03322. doi: 10.1016/j.heliyon.2020.e03322 32051879 PMC7002885

[17] Valdovinos VS Juárez-Loya A Ramos-Lira L González-Forteza C Valdez-Santiago R . International Trauma Questionnaire (ITQ): Psychometric properties of the Spanish-language version in a clinical sample of Mexican women. J Aggress Maltreatment Trauma. (2023) 32:935–49. doi: 10.1080/10926771.2023.2171828 37339054

[18] Miaja Avila M Moral de la Rubia J Fonseca A Cruz Ramos M Villarreal Garza C Becerril Gaitán A . Factor structure, internal consistency and distribution of Female Sexual Function Index among Mexican women with early diagnostic of breast cancer. Psicooncología Investig Clínica Biopsicosocial En Oncol. (2021) 18:293–316. doi: 10.5209/psic.77755

[19] Téllez A Almaraz-Castruita DA Valdez A Juárez-García DM de Jesús Sánchez-Jáuregui T Hinojosa Fernández R . Validating the Spanish Adverse Childhood Experiences International Questionnaire (ACE-IQ): A Mexican analysis. J Aggress Maltreatment Trauma. (2023) 32:918–34. doi: 10.1080/10926771.2022.2144788 37339054

[20] Posit team . RStudio: Integrated Development Environment for R. Boston, MA: Posit Software, PBC (2025). Available online at: http://www.posit.co/ (Accessed December 15, 2025).

[21] Bornefeld-Ettmann P Steil R Lieberz KA Bohus M Rausch S Herzog J . Sexual functioning after childhood abuse: The influence of post-traumatic stress disorder and trauma exposure. J Sex Med. (2018) 15:529–38. doi: 10.1016/j.jsxm.2018.02.016 29550460

[22] Hill K Robinson LE Yadav MR Heidel JR Nelis M Kulkarni R . The gynecological sequelae of sexual violence in adolescence in the United States: A scoping review. Int J Gynecol Obstet Off Organ Int Fed Gynecol Obstet. (2025) 169:1065–92. doi: 10.1002/ijgo.16112 39950711

[23] Carvalho J Vieira AL Nobre P . Latent structures of female sexual functioning. Arch Sex Behav. (2012) 41:907–17. doi: 10.1007/s10508-011-9865-7 22170444

[24] Tetik S Yalçınkaya Alkar Ö . Vaginismus, dyspareunia and abuse history: A systematic review and meta-analysis. J Sex Med. (2021) 18:1555–70. doi: 10.1016/j.jsxm.2021.07.004 34366265

[25] Bryant RA Felmingham KL Malhi G Andrew E Korgaonkar MS . The distinctive neural circuitry of complex posttraumatic stress disorder during threat processing. Psychol Med. (2021) 51:1121–8. doi: 10.1017/S0033291719003921 31910918

[26] McBenedict B Petrus D Pires MP Pogodina A Arrey Agbor DB Ahmed YA . The role of the insula in chronic pain and associated structural changes: An integrative review. Cureus. (2024) 16:e58511. doi: 10.7759/cureus.58511 38770492 PMC11103916

[27] Neugebauer V . Amygdala physiology in pain. Handb Behav Neurosci. (2020) 26:101–13. doi: 10.1016/b978-0-12-815134-1.00004-0 33889063 PMC8059430

[28] Bryant RA Tran J Williamson T Korgaonkar MS . Neural processes during response inhibition in complex posttraumatic stress disorder. Depress Anxiety. (2022) 39:307–14. doi: 10.1002/da.23235 34964209

[29] Hwang K Bertolero MA Liu WB D’Esposito M . The human thalamus is an integrative hub for functional brain networks. J Neurosci Off J Soc Neurosci. (2017) 37:5594–607. doi: 10.1523/JNEUROSCI.0067-17.2017 28450543 PMC5469300

[30] Huffman N Shih CH Cotton AS Lewis TJ Grider S Wall JT . Association of age of adverse childhood experiences with thalamic volumes and post-traumatic stress disorder in adulthood. Front Behav Neurosci. (2023) 17:1147686. doi: 10.3389/fnbeh.2023.1147686 37283956 PMC10239841

[31] Karsten MDA Wekker V Bakker A Groen H Olff M Hoek A . Sexual function and pelvic floor activity in women: the role of traumatic events and PTSD symptoms. Eur J Psychotraumatol. (2020) 11:1764246. doi: 10.1080/20008198.2020.1764246 33029306 PMC7473031

[32] Jakob JMD Lamp K Rauch SAM Smith ER Buchholz KR . The impact of trauma type or number of traumatic events on PTSD diagnosis and symptom severity in treatment seeking veterans. J Nerv Ment Dis. (2017) 205:83–6. doi: 10.1097/NMD.0000000000000581 28129258

[33] Olaya B Alonso J Atwoli L Kessler RC Vilagut G Haro JM . Association between traumatic events and post-traumatic stress disorder: results from the ESEMeD-Spain study. Epidemiol Psychiatr Sci. (2015) 24:172–83. doi: 10.1017/S2045796014000092 24565167 PMC4143480

[34] Karatzias T Shevlin M Fyvie C Hyland P Efthymiadou E Wilson D . Evidence of distinct profiles of posttraumatic stress disorder (PTSD) and complex posttraumatic stress disorder (CPTSD) based on the new ICD-11 Trauma Questionnaire (ICD-TQ). J Affect Disord. (2017) 207:181–7. doi: 10.1016/j.jad.2016.09.032 27723542

[35] Galovski TE Werner KB Weaver TL Morris KL Dondanville KA Nanney J . Massed cognitive processing therapy for posttraumatic stress disorder in women survivors of intimate partner violence. Psychol Trauma Theory Res Pract Policy. (2022) 14:769–79. doi: 10.1037/tra0001100 34472941

[36] Meis LA Alpert E Peterson ZD Livingston WS Blain L Galovski TE . Change in sexual distress during cognitive processing therapy: Characterization and baseline predictors. J Affect Disord. (2026) 393:120389. doi: 10.1016/j.jad.2025.120389 41076157 PMC13284763

[37] Badour CL Cox KS Goodnight JRM Flores J Tuerk PW Rauch SAM . Sexual desire among veterans receiving prolonged exposure therapy for PTSD: Does successful PTSD treatment also yield improvements in sexual desire? Psychiatry. (2020) 83:70–83. doi: 10.1080/00332747.2019.1672439 31577915 PMC7083685

[38] van Woudenberg C Voorendonk EM Tunissen B van Beek VHF Rozendael L Van Minnen A . The impact of intensive trauma-focused treatment on sexual functioning in individuals with PTSD. Front Psychol. (2023) 14:1191916. doi: 10.3389/fpsyg.2023.1191916 37614489 PMC10442952

[39] Hu JH Ma YQ Zhou Y Wang SB Jia FJ Hou CL . Efficacy of psychological interventions for complex post-traumatic stress disorder in adults exposed to complex traumas: A meta-analysis of randomized controlled trials. J Affect Disord. (2025) 380:515–26. doi: 10.1016/j.jad.2025.03.153 40154799

[40] Schaug JP Møller L Reinholt N Illum DB Græbe FL Mikkelsen LB . Psychotherapies for adults with complex presentations of PTSD: a clinical guideline and five systematic reviews with meta-analyses. BMJ Ment Health. (2025) 28:e301158. doi: 10.1136/bmjment-2024-301158 40234083 PMC12004466

